# Clinical impact of primary tumour location, early tumour shrinkage, and depth of response in the treatment of metastatic colorectal cancer with first-line chemotherapy plus cetuximab or bevacizumab

**DOI:** 10.1038/s41598-020-76756-1

**Published:** 2020-11-13

**Authors:** Tamotsu Sagawa, Yasushi Sato, Masahiro Hirakawa, Kyoko Hamaguchi, Akira Fukuya, Koichi Okamoto, Hiroshi Miyamoto, Naoki Muguruma, Koshi Fujikawa, Yasuo Takahashi, Tetsuji Takayama

**Affiliations:** 1 Department of Gastroenterology, Hokkaido Cancer Center, Sapporo, Hokkaido Japan; 2 Department of Community Medicine for Gastroenterology and Oncology, Tokushima University Graduate School of Biomedical Sciences, Tokushima, Japan; 3 Department of Gastroenterology and Oncology, Tokushima University Graduate School of Biomedical Sciences, Tokushima, Japan

**Keywords:** Chemotherapy, Targeted therapies, Cancer, Colorectal cancer

## Abstract

The primary tumour location is an important prognostic factor for previously untreated metastatic colorectal cancer (mCRC). However, the predictive efficacies of primary tumour location, early tumour shrinkage (ETS), and depth of response (DpR) on mCRC treatment has not been fully evaluated. This study aimed to investigate the predictive efficacies of these traits in mCRC patients treated with first-line 5-fluorouracil-based chemotherapy plus biologic agents, namely, cetuximab and bevacizumab. This was a retrospective analysis of the medical records of 110 patients with pathology-documented unresectable mCRC. Patients with left-sided mCRC receiving any first-line regimen showed better overall survival (OS) than those with right-sided mCRC [33.3 vs 16.3 months; hazard ratio (HR) 0.44; 95% confidence interval (CI) 0.27–0.74; *p* < 0.001]. In patients with left-sided tumours, treatment with chemotherapy plus cetuximab yielded longer OS than chemotherapy plus bevacizumab (50.6 vs 27.8 months, HR 0.55; 95% CI 0.32–0.97; *p* = 0.0378). mCRC patients with ETS and high DpR showed better OS than those lacking ETS and with low DpR (33.5 vs 19.6 months, HR 0.50, 95% CI 0.32–0.79, *p* = 0.023 and 38.3 vs 19.0 months, HR 0.43, 95% CI 0.28–0.68, *p* < 0.001, respectively). Moreover, ETS and/or high DpR achieved in patients with right-sided mCRC receiving chemotherapy plus cetuximab were associated with significantly better OS than in those lacking ETS and with low DpR (34.3 vs 10.4 months, HR 0.19, 95% CI 0.04–0.94, *p* = 0.025 and 34.3 vs 10.4 months, HR 0.19, 95% CI 0.04–0.94, *p* = 0.0257, respectively). Taken together, our study demonstrates that primary tumour location is not only a well-known prognostic factor but also a relevant predictive factor in patients with mCRC receiving chemotherapy plus cetuximab. Additionally, both ETS and DpR could predict treatment outcomes and also potentially guide cetuximab treatment even in right-sided mCRCs.

## Introduction

Colorectal cancer (CRC) is the second leading cause of cancer-related deaths worldwide^1^. However, with advances in chemotherapeutic and biologic agents, diagnosis, and ablative techniques, the survival of patients with unresectable metastatic CRC (mCRC) has improved from 12 to over 30 months^2^. The standard first-line therapy for mCRC includes 5-fluorouracil plus folic acid combined with irinotecan (FOLFIRI) or oxaliplatin (FOLFOX)^3^. Further, in previous Phase-III randomised trials, compared to the FOLFIRI or FOLFOX regimen, administration of either an anti-epidermal growth factor receptor (EGFR) monoclonal antibody (cetuximab and panitumumab) or an anti-vascular endothelial growth factor (VEGF) antibody (bevacizumab) showed favourable OS, progression-free survival (PFS), and overall response rate (ORR) in the patients with CRC^4–6^. However, when the efficacies of combination therapies of cetuximab or bevacizumab with FOLFIRI or FOLFOX were compared, the outcomes were controversial. The FIRE-3 study randomised mCRC patients with *KRAS* exon 2 wild-type (wt) status into either FOLFIRI plus cetuximab or bevacizumab groups and the median OS was found to be better upon treatment with FOLFIRI plus cetuximab^7^. In contrast, Venook et al. in their CALGB/SWOG 80,405 study observed no significant difference in OS upon administration of cetuximab vs bevacizumab compared to the FOLFOX or FOLFIRI regimen for the treatment of patients with *KRA*S wt, advanced CRC or mCRC^8^. Therefore, a detailed analysis is required to comprehend the efficacy of either treatment modalities.

Several studies have embarked upon identifying prognostic and predictive factors for targeted therapy using anti-EGFR antibodies in patients with mCRC. The anti-EGFR antibody did not show any clinical benefits for patients with any RAS mutation^9,10^. Therefore, it is essential to confirm the mutational status of *RAS* before administering anti-EGFR therapy to patients with mCRC. Furthermore, while the V600E *BRAF* mutation is a negative prognostic marker for mCRC, its utility in predicting response to anti-EGFR therapy is still uncertain^11^. The HER2 status may impact the response to anti-EGFR therapies since its activation substitutes EGFR dependence in a subset of mCRC patients, thus serving as a potentially negative predictor of the beneficial effects of anti-EGFR therapy^12^.

In contrast, the primary tumour location has been shown to predict prognosis in patients with previously untreated mCRC^13^, supported by post hoc analyses of large randomised controlled trials and meta-analyses. Holch et al.^14^ reported that in patients receiving bevacizumab and cetuximab, tumours originating in the right-side were associated with higher mortality than left-sided tumours. Additionally, several studies found the primary tumour location to be predictive of response to anti-EGFR therapy, although only in patients with a left-sided tumour harbouring a *RAS* wt status^15–17^. Furthermore, in the pooled analysis of six randomised trials, mCRC patients with left-sided tumours harbouring *RAS* wt status showed favourable ORR, OS, and PFS than those with right-sided tumours, and the analyses predicted better response upon treatment with chemotherapy plus anti-EGFR therapy than chemotherapy alone or chemotherapy plus anti-VEGF therapy^18^. Taken together, these findings suggest that the location of tumours on the left side can help predict the efficacy of anti-EGFR antibodies in mCRC patients with *KRAS* wt status.

Recent retrospective analyses of clinical trial data have included early tumour shrinkage (ETS) and depth of response (DpR) as measures for assessing tumour response^3,19,20^. While ETS offers an early indication of sensitivity to treatment and is associated with improved PFS and OS irrespective of the treatment received, the DpR reveals the maximum tumour shrinkage achieved, which relates to the post-progression survival. However, their clinical relevance has not been firmly established, and there are limited data on these response assessments according to primary tumour location.

The studies presented above are retrospective analyses of randomised clinical trials, majorly conducted in western nations, and their outcomes cannot be generalised to patients in actual clinics. Therefore, here, we investigated the prognostic and predictive efficacy of primary tumour location and the impact of ETS and/or DpR on therapeutic outcomes in a cohort of patients with mCRC treated with first-line chemotherapy plus bevacizumab or cetuximab in the real-world setting in a Japanese population.

## Results

### Patient characteristics

**Figure 1:**
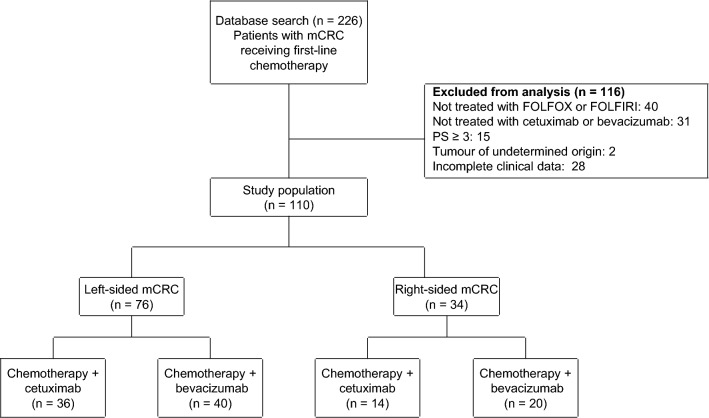
Flow diagram indicating patient details. *mCRC* metastatic colorectal cancer.

Based on the eligibility criteria, 110 patients with mCRC, who received first-line treatment were included in the study (Fig. 1). The patient characteristics based on the location of the primary tumour, are summarised in Table 1. There were 76 (69.1%) patients with left-sided and 34 (30.9%) with right-sided mCRC. Overall, the clinical characteristics of the patients, such as *KRAS* (*RAS*) status, tumour condition (advance or recurrence), comorbidity (with or without), and primary tumour status (resection or no resection), were comparable in the left-sided and right-sided tumours. However, significant differences were observed in the histological statuses, with 72 of the 76 left-sided tumours classified as adenocarcinoma (94.7%), whereas 10 of the 34 right-sided tumours were mucinous (29.4%). Further, the oxaliplatin-based regimen was the most widely used chemotherapy (70 of 110 patients, 63.6%), although no significant differences were observed between irinotecan-based and oxaliplatin-based regimens for the treatment of left- and right-sided tumours. Additionally, bevacizumab-containing chemotherapy was preferred over cetuximab-containing regimen (60 of 110 (54.5%) vs 50 of 110 (45.5%) patients, respectively). However, no significant differences were noted for the use of biological agents between left- and right-sided tumours. Furthermore, 46 of 60 patients (76.7%) receiving bevacizumab had *KRAS* mutation.

**Table 1:** Patient characteristics and treatments grouped based on primary tumour sites.

	All (n = 110)	Left-sided (n = 76)	Right-sided (n = 34)	*p* value
Age (years), median (range)	65 (23–84)	63 (30‒82)	67 (23‒84)	
**Gender**				0.064
Male, n (%)	54 (49)	42 (55.3)	12 (32.4)	
Female, n (%)	56 (51)	34 (44.7)	22 (67.6)	
**Comorbidity**				0.889
Yes	7 (6.4)	5(6.6)	2 (5.9)	
No	103 (93.6)	71 (93.4)	32 (94.1)	
**Histologic type**				0.001
Adenocarcinoma, n (%)	96 (87.3)	72 (94.7)	24 (70.6)	
Mucinous, n (%)	14 (12.7)	4 (5.3)	10 (29.4)	
**KRAS (RAS) status**^**a**^				0.144
*KRAS* (*RAS*) wild/mutant, n	64 (11)/46	48 (7) / 28	16 (4) / 18	
**Condition**				0.227
Advanced, n (%)	85 (77.3)	56 (73.7)	29 (85.3)	
Recurrent, n (%)	25 (22.3)	20 (26.3)	5 (14.7)	
**Primary tumour**				0.831
Resection, n (%)	70 (63.6)	49 (64.5)	21 (61.8)	
No Resection, n (%)	40 (36.4)	27 (35.5)	13 (38.2)	
**Doublet chemotherapy**				0.392
Oxaliplatin base, n (%)	70 (63.6)	46 (60.5)	24 (70.6)	
Irinotecan base, n (%)	40 (36.4)	30 (39.5)	10 (29.4)	
**Type of biological agent**				
Bevacizumab, n (%)	60 (54.5)	40 (52.6)	20 (58.8)	0.546
Cetuximab, n (%)	50 (45.5)	36 (47.4)	14 (41.2)	

^a^All patients receiving cetuximab were confirmed for *KRAS* wt or *RAS* wt.

### Efficacy of outcome based on primary tumour location and molecular-targeted agents

The ORR observed for all the patients was 66 (60%), of which 4 showed complete response (3.6%) and 62 showed partial response (54.6%). Left-sided tumours showed significantly higher ORR than the right-sided tumours (67.1% vs 44.1%; *p* = 0.003, Table 2). Further, no significant differences were observed upon treatment with chemotherapy plus cetuximab or bevacizumab in patients with either left- or right-sided tumours (Table 2). Moreover, the ORR did not vary upon treatment with cetuximab or bevacizumab (66.0% vs 55.5%, Table S1). Additionally, while the difference between the ORR of left- and right-sided tumours upon treatment with chemotherapy plus bevacizumab was not significant (*p* = 0.596), treatment with cetuximab conferred better ORR in patients with left-sided tumours (77.8%) than in those with right-sided tumours (35.7%, *p* = 0.008, Table S1).

**Table 2:** Efficacy for the first-line treatment in patients with mCRC stratified based on the molecular-target agent and tumour location.

	Left-sided mCRC (n = 76)			Right-sided mCRC (n = 34)		
	All	Cetuximab (n = 36)	Bevacizumab (n = 40)	All	Cetuximab (n = 14)	Bevacizumab (n = 20)
CR	3 (4.3%)	3 (8.3%)	0 (0.0%)	1 (2.9%)	1 (7.1%)	0 (0.0%)
PR	48* (63.2%)	25 (69.4%)	23 (57.5%)	14* (41.2%)	4 (28.6%)	10 (50.0%)
SD	22 (28.9%)	6 (16.7%)	16 (40.0%)	16 (47.1%)	6 (42.9%)	10 (50.0%)
PD	3 (3.9%)	2 (5.6%)	1 (2.5%)	3 (8.8%)	3 (21.4%)	0 (0.0%)
ORR	51* (67.1%)	28 (77.8%)	23 (57.5%)	15* (44.1%)	5 (35.7%)	10 (50%)

*CR* complete response, *PD* progressive disease, *PR* partial response, *RD* recommended dose, *SD* stable disease, *ORR* overall response.

*p value for difference between tumour side < 0.05.

### Assessment of progression-free survival and overall survival based on the primary tumour location

Overall, the median PFS, median OS and median follow-up duration of all the patients combined was 11.4, 27.8, and 25.1 (range 1.8–103.2) months, respectively.

**Figure 2:**
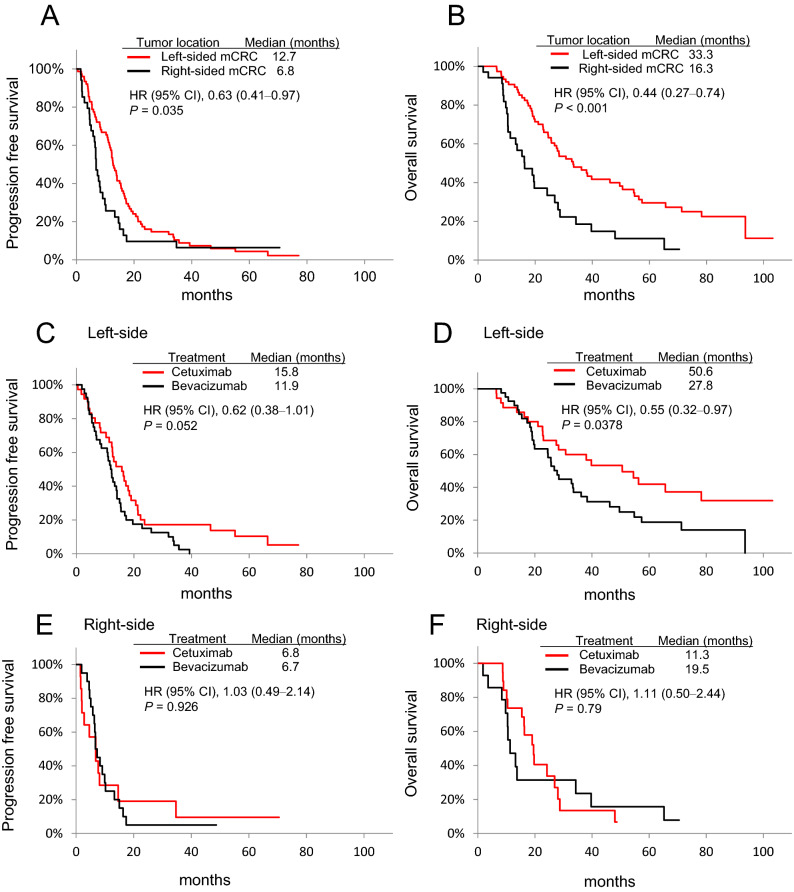
Kaplan–Meier analyses of mCRC samples based on tumour location. (**A**) Progression-free survival (PFS) and (**B**) overall survival (OS) for all the patients; (**C**) PFS and (**D**) OS in patients with left-sided mCRCs treated with chemotherapy plus cetuximab or bevacizumab; (**E**) PFS and (**F**) OS in patients with right-sided mCRCs treated with chemotherapy plus cetuximab or bevacizumab. *CI* confidence interval, *HR* hazard ratio.

Comparison of the primary tumour location suggested significantly better median PFS (12.7 vs 6.8 months; HR 0.63; 95% CI 0.41‒0.97; *p* = 0.035) (Fig. 2A), and median OS (33.3 vs 16.3 months; HR 0.44; 95% CI 0.27‒0.74; *p* < 0.001) (Fig. 2B) in patients with left-sided tumours than in those with right-sided tumours, respectively. Moreover, treatment of patients with left-sided tumours using cetuximab significantly prolonged the median OS than did bevacizumab (50.6 vs 27.8 months; HR 0.55; 95% CI 0.32‒0.97; *p* = 0.0378), although the difference in PFS was not significant (15.8 vs 11.9 months; HR 0.62; 95% CI 0.38‒1.01; *p* = 0.052) (Fig. 2C,D). Furthermore, treatment of patients with right-sided tumours using either cetuximab or bevacizumab did not follow the trend, and the differences in median PFS (6.8 vs 6.7 months; HR 1.03; 95% CI 0.49‒2.14; *p* = 0.926) and median OS (11.3 vs 19.5 months; HR 1.11; 95% CI 0.50‒2.44; *p* = 079) were not significant. (Fig. 2E,F).

### Assessment of progression-free survival and overall survival based on the biological therapy group

Next, we examined the PFS and OS of patients treated with cetuximab or bevacizumab (Fig. S1A,B). The PFS did not differ for patients treated with cetuximab (n = 50) or bevacizumab (n = 60) (12.6 vs 10.2 months, respectively; HR 0.68; 95% CI 0.45‒1.02; *p* = 0.061), and the OS followed a similar trend (38.0 vs 25.6 months, respectively; HR 0.65; 95% CI 0.41‒1.01; *p* = 0.0584). Further, we assessed the PFS and OS based on primary tumour location in patients treated with these agents (Fig. S1C-F). Our analysis suggests that patients with left-sided tumours showed better median OS and PFS than those with right-sided tumours upon treatment with cetuximab (50.6 vs 11.3 months; HR 0.34; 95% CI, 0.16‒0.71; *p* = 0.0026), although the difference in PFS was not significant (15.8 vs 6.8 months; HR 0.60; 95% CI 0.30‒1.18; *p* = 0.136) (Fig. S1C,D). Furthermore, treatment with bevacizumab resulted in better median PFS in patients with left-sided tumours than in those with right-sided tumours (11.9 vs 6.7 months, respectively; HR 0.70; 95% CI 0.40‒1.22; *p* = 0.234), along with better median OS (27.8 vs 19.0 months, respectively; HR 0.48; 95% CI 0.25‒0.90; *p* = 0.0199) (Fig. S1E, F).

### Predictive value of the primary tumour location, ETS, and DpR

**Figure 3:**
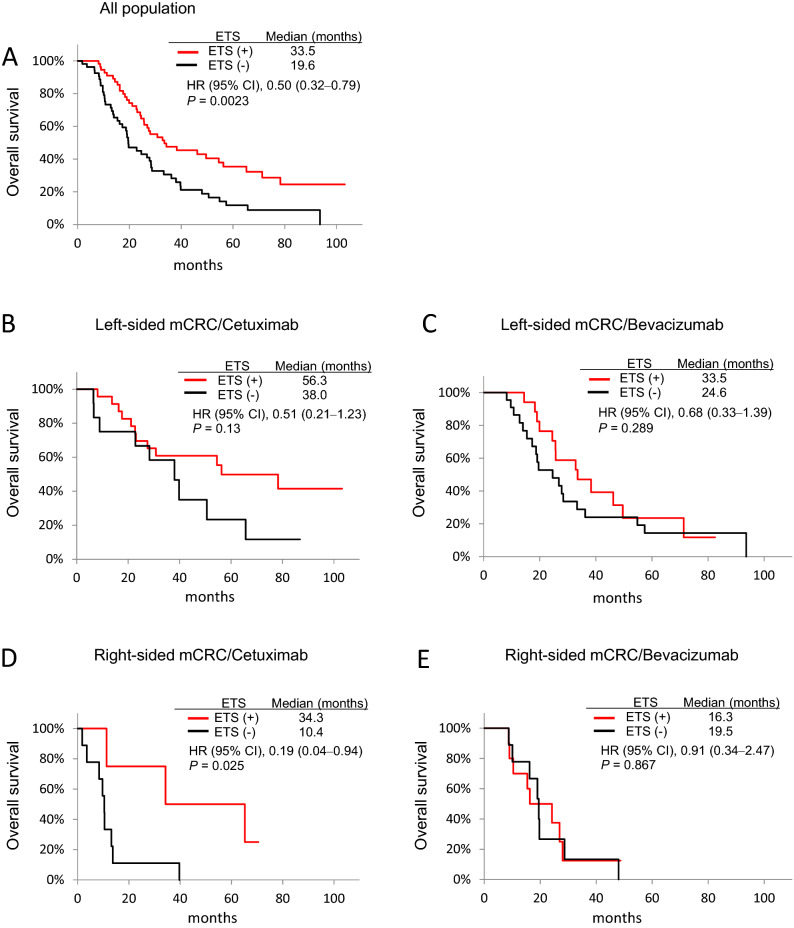
Kaplan–Meier analyses of mCRC based on ETS. (**A**) OS in all patients receiving chemotherapy plus cetuximab or bevacizumab; (**B**) OS in patients with left-sided mCRCs receiving chemotherapy plus cetuximab; (**C**) OS in patients with left-sided mCRCs receiving chemotherapy plus bevacizumab; (**D**) OS in patients with right-sided mCRCs receiving chemotherapy plus cetuximab; and (**E**) OS in patients with right-sided mCRCs receiving chemotherapy plus bevacizumab. *CI* confidence interval, *HR* hazard ratio.

The data for tumour shrinkage from baseline till the 8th week was available for 110 patients with mCRC, and the patient outcome, including ETS and DpR, based on primary tumour location is summarised in Table 3. Seventy-one patients (64.5%) experienced ETS and showed significantly better OS than those who did not experience ETS (33.5 vs 19.6 months; HR 0.50; 95% CI 0.32‒0.79; *p* = 0.0023) (Fig. 3A).

**Table 3:** Early tumour shrinkage (EST) and depth of response (DpR) for the first-line treatment in patients with mCRC stratified based on the molecular-target agent and tumour location.

	Total (n = 110)	Left-sided mCRC (n = 76)			Right-sided mCRC (n = 34)		
		All	Cetuximab (n = 36)	Bevacizumab (n = 40)	All	Cetuximab (n = 14)	Bevacizumab (n = 20)
EST (+), n (%)	71 (64.5)	56 (73.7)	23 (63.9)	33 (52.5)	15 (44.1)	5 (35.7)	10 (50.0)
DpR, median, (range)	− 30.0 (− 100 to + 45)	− 32.0 (− 100 to + 30)	− 43.5* (− 100 to + 30)	− 30.0* (− 64 to + 25)	− 27.5 (− 100 to + 45)	− 9.6 (− 100 to + 45)	− 33.0 (− 58 to + 3)

*EST* early tumour shrinkage, *DpR* depth of response.

*p value for difference between cetuximab and bevacizumab  < 0.05.

In all the treatment arms, patients with left-sided tumours experienced ETS more than those with right-sided tumours (56 (73.7%) vs 15 (44.1%), respectively). Further, patients with left-sided tumours treated with cetuximab showed high ETS than those treated with bevacizumab (63.9% vs 52.5%, *p* = 0.065, Table 3), and patients with ETS also showed better OS, irrespective of treatment with either of the drugs (Fig. 3B,C).

In contrast, patients with right-sided tumours showed higher ETS when treated with bevacizumab than with cetuximab (50% vs 35.7%, *p* = 0.4, Table 3). However, patients treated with cetuximab that showed ETS presented with significantly better OS than those that did not experience ETS (34.3 vs 10.4 months; HR 0.19; 95% CI, 0.04‒0.94; *p* = 0.025) (Fig. 3D), although the difference in OS was not significant (16.3 vs 19.5 months; HR 0.91; 95% CI 0.34‒2.47; *p* = 0.867) in patients with right-sided tumours treated with bevacizumab (Fig. 3E).

**Figure 4:**
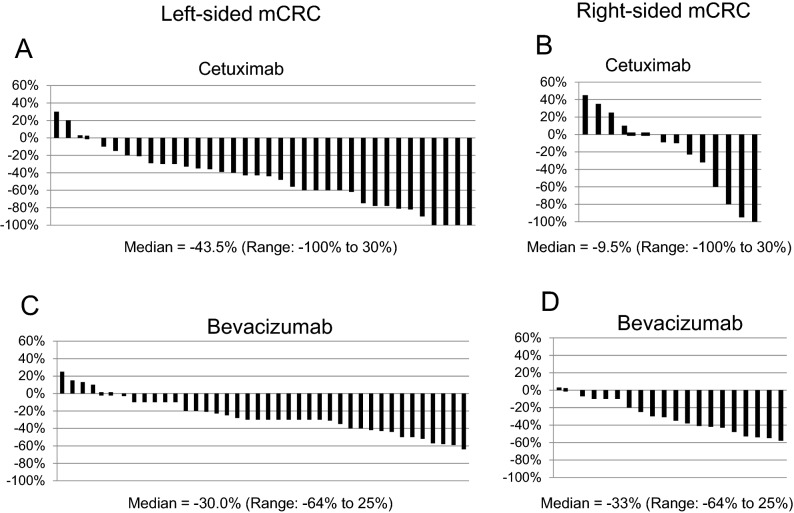
Comparison of waterfall plots showing the distribution of DpR. Waterfall plot of DpR in (**A**) patients with left-sided mCRCs receiving chemotherapy plus cetuximab; (**B**) patients with right-sided mCRCs receiving chemotherapy plus cetuximab; (**C**) patients with left-sided mCRCs receiving chemotherapy plus bevacizumab; and (**D**) patients with right-sided mCRCs receiving chemotherapy plus bevacizumab.

Next, the median DpR in patients with left-sided mCRCs receiving cetuximab and bevacizumab was − 43.5% (range − 100 to 30) and − 30.0% (range − 64 to 25), respectively (*p* = 0.001). On the other hand, the median DpR in patients with right-sided mCRCs receiving cetuximab and bevacizumab was − 9.6% (range − 100 to 45) and − 33% (range − 58 to 3), respectively (*p* = 0.42) (Table 3). The DpR of individual patients with left- and right-sided mCRCs treated with either cetuximab or bevacizumab are shown in Fig. 4. The maximal DpR was observed in a few patients with left- and right-sided mCRCs, and particularly, 5 patients who received chemotherapy plus cetuximab showed 100% reduction, and 2 of them underwent complete (R0) resection (Fig. 4A,B).

**Figure 5:**
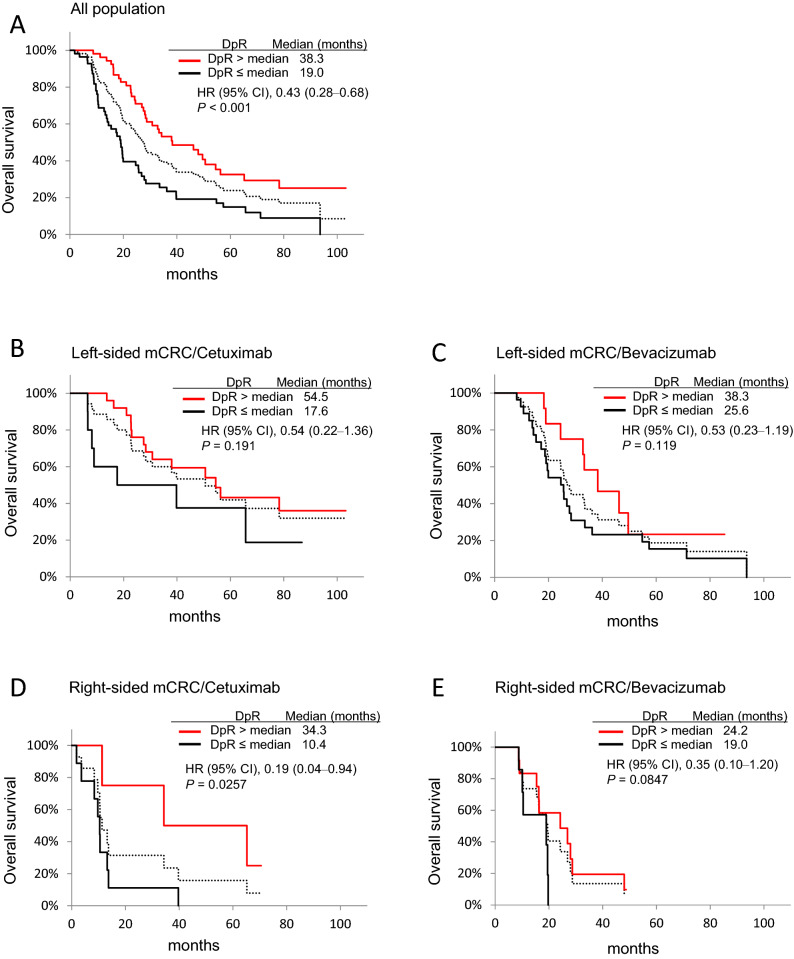
Kaplan–Meier analyses of mCRC samples based on DpR. (**A**) OS in all patients receiving chemotherapy plus cetuximab or bevacizumab; (**B**) OS in patients with left-sided mCRCs receiving chemotherapy plus cetuximab; (**C**) OS in patients with left-sided mCRCs receiving chemotherapy plus bevacizumab; (**D**) OS in patients with right-sided mCRCs receiving chemotherapy plus cetuximab; and (**E**) OS in patients with right-sided mCRCs receiving chemotherapy plus bevacizumab. Median DpRs were calculated as -30 for A, -32 for B and C, and -27.5 for D and E, respectively. *CI* confidence interval, *HR* hazard ratio. Broken line, all population.

To evaluate the relationship between patient DpR and survival, we classified DpR into high and low groups based on the median DpR value (Fig. 5). The median DpR of all the treatment population was − 30 (range − 100 to 45, Table 3), and patients with high DpR showed better OS than those with low DpR (38.3 vs 19.0 months; HR 0.43; 95% CI 0.28‒0.68; *p* < 0.001) (Fig. 5A). In the patients with right-sided tumours, the high DpR group showed better OS than the low DpR group when treated with cetuximab (34.3 vs 10.4 months, *p* = 0.0257) (Fig. 5D).

## Discussion

Here, we enrolled a cohort of consecutive patients with mCRC in real-world clinical setting, and assessed factors known to affect their prognosis and treatment outcomes, such as primary tumour location, biologic therapy (bevacizumab or cetuximab), and the more recently proposed indicators, EST and DpR.

Our findings support the notion that primary tumour location is an important prognostic factor in mCRC and can help guide treatment decisions on the first-line regimen based on the findings that clinical benefits for cetuximab were observed in patients with left-sided mCRC. In addition, ETS (≥ 20% at week 8) and high DpR during treatment with first-line agents were associated with favourable outcomes in patients with mCRC, further supporting the use of these endpoints clinically. Furthermore, our study raises the possibility of a predictive relevance of ETS and DpR in guiding targeted therapy (cetuximab) in patients with right-sided mCRC.

First, our study demonstrated that patients with left-sided mCRC receiving chemotherapy plus cetuximab or bevacizumab showed better ORR, PFS, and OS than those with right-sided mCRC (Table 2; Fig. 2A,B), reconfirming previous evidence that primary tumour location serves as a prognostic factor for patients treated with any first-line regimen^13,14,17,18^. Further, we observed an association between the primary tumour location and treatment with cetuximab, where patients with left-sided tumours showed better survival outcomes when treated with cetuximab than with bevacizumab (Fig. 2C,D). However, right-sided tumours responded differently and showed no significant difference in survival outcomes upon treatment with bevacizumab or cetuximab (Fig. 2E,F). Moreover, these observations were found to be corroborated by several randomised clinical trials that reported tumours on the left side as predictors of response to cetuximab, while those on the right side as non-responsive to cetuximab despite the *RAS* wt status^15,16,21,22^.

Together, these observations suggest that mCRC is a heterogeneous disease with the primary tumours originating from different areas of the colon showing molecular differences^23^. According to the CRC Subtyping Consortium, consensus molecular subtype 2 (CMS2) is predominantly distributed in left-sided tumours and shows activated EGFR-dependent signalling defined by the over-expression of EGFR. Although right-sided tumours showed high proportions of the microsatellite unstable/immune CMS1 and the metabolic CMS3 subtypes^24^, these differences could be among the factors contributing to differential responses to treatment with cetuximab based on tumour location^25^. However, the main genetic drivers and crucial factors contributing to the vast differences remain largely unknown.

Further, since *RAS* is mutated (*RAS* mt) in about 55% of the mCRC cases, these patients need to be treated with bevacizumab^26^. However, the majority of recently published studies with randomised trials have focused on the *RAS* wt subpopulation alone; hence, data on treatment with bevacizumab for *RAS* mt mCRC involving the two primary tumour locations are limited^16,27,28^. Moreover, while several clinical studies have suggested that the tumour location does not predict benefit from bevacizumab treatment in patients with mCRC harbouring *KRAS* wt^16,29^ and undetermined *RAS* status^30^, other reports indicate better outcomes in left- than right-sided mCRC, with unselected *RAS* status, treated with bevacizumab^13,17,30^. Our analysis suggests that patients treated with bevacizumab had the *RAS* mt-mCRC predominantly, and left-sided mCRCs were associated with better PFS and OS than right-sided mCRCs (Fig. S1E,F). Therefore, these findings suggest that the primary tumour location is closely associated with outcomes even in patients with *RAS* mt status treated with chemotherapy plus bevacizumab. However, further investigations would be required to determine whether the primary tumour location and treatment with chemotherapy plus bevacizumab is associated with different effects, especially in patients with *RAS*-mt mCRC.

Next, our analysis validated the previous observation that patients with ETS and high DpR show an improved OS than those without ETS and with low DpR^3,19,20^. Further, we observed that the frequency of ETS was high in patients with left-sided mCRC than in those with right-sided mCRC. Moreover, patients with left-sided mCRC receiving cetuximab-based regimen showed a high frequency of ETS (Table 3), which may result in favourable OS than in those who did not show ETS and other groups (Fig. 3). Although limited data are available, especially comparing biologic agents, recent retrospective analysis of the PEAK trail^31^, revealed that a high proportion of patients with left-sided mCRC achieved ETS and better OS when treated with panitumumab than bevacizumab. Furthermore, the DpR is reported to be associated with a maximal response, which in turn relates to post-progression survival in patients^20^. Consistent with this, we observed a high DpR in patients with left-sided mCRCs receiving cetuximab, along with a complete reduction in tumour content in 4 of 36 patients (Fig. 4A) and significantly better OS in the high DpR than in the low DpR groups (Fig. 5A). Additionally, although the median DpR was low in the patients with right-sided mCRCs receiving cetuximab (Table 3), few patients had a high response rate (Fig. 4B), and those with high DpR showed significantly better OS than those with low DpR (Fig. 5D). Interestingly, our analysis suggested that patients with ETS also presented with significantly better OS than those without ETS (Fig. 3A) and with right-sided mCRCs receiving cetuximab (Fig. 3D), consistent with the findings of the PEAK trial^31^. Taken together, these findings suggest that the ETS/DpR may help identify a subgroup of patients with right-sided tumours who may achieve improved outcomes. Further, the guidelines of the NCCN clinical practice for Colon cancer, version 4. 2020, recommend anti-EGFR monoclonal antibody for the treatment of patients with *KRAS/NRAS* wt status and left-sided mCRC alone. However, our analysis indicated that administering bevacizumab might not be the only treatment option in patients with right-sided mCRCs harbouring *RAS* wt and that cetuximab might be considered in a certain subtype of mCRC with ETS and/or high DpR. Moreover, the molecular markers influencing clinical benefit in patients with right-sided mCRCs treated with anti-EGFR therapy would be worth investigating.

There are several limitations to the present investigation. First, the patient sample size was small, particularly, that of the right-sided tumours, although the comparisons yielded statistical significance that strengthened our analysis.

Second, because this was a retrospective study, limited baseline characterisation and the heterogeneity of treatments in the study seemed to be a limitation; however, patient characteristics and treatment groups were almost comparable in left-sided and right-sided tumours. Moreover, heterogeneity as regards several molecular markers for the prognosis of mCRC, such as the unavailability of the *BRAF* status and microsatellite instability, could have altered the results.

Third, a large proportion of the patients treated with bevacizumab were mutant for *KRAS/RAS*, and this might have affected our analysis, especially the comparison of efficacy between left- and right-sided tumours. However, this effect could be of less impact since the *KRAS* (*RAS*) status of tumours was well balanced between the two cohorts. Finally, we may need to consider that treatment with bevacizumab may be less active in *KRAS*/*RAS* mutant mCRC patients, although such data are rare^32^.

In conclusion, our study supports the notion that the primary tumour location is a well-known prognostic factor and also a relevant predictive factor in patients with mCRCs receiving cetuximab. Further, our analysis indicates that chemotherapy plus cetuximab may serve as the preferred option for the treatment of patients with *RAS* wt left-sided tumours to provide better OS, while the right-sided tumours presented with poor prognosis with all treatment measures. However, mCRC patients with right-sided tumours harbouring *RAS* wt status, who received chemotherapy plus cetuximab and achieved ETS and/or high DpR, showed better clinical benefit than those without ETS and low DpR. These results suggest that in patients with *RAS* wt right-sided mCRCs, bevacizumab might not be the only treatment option, and cetuximab may be considered in a subtype of mCRC, wherein ETS and/or high DpR can be expected.

## Methods

### Patients and study design

In this observational study, the medical records of 110 of 226 patients with pathology-documented metastatic Stage IV CRC, who underwent treatment between January 2011 and December 2016 at the Department of Gastroenterology, Hokkaido Cancer Center, Sapporo, Japan, were analysed retrospectively. The study was reviewed and approved by the Ethics Committee of Hokkaido Cancer Centre (No. 29-60), and the need for informed consent was waived. The patient records were anonymised and de-identified before analysis. All procedures performed in studies involving human participants were in accordance with the ethical standards of the institution and/or national research committee and with the 1964 Helsinki declaration and its later amendments or comparable ethical standards.

The inclusion criteria were as follows: (a) treatment with either FOLFOX or FOLFIRI plus cetuximab or bevacizumab; (b) patients above 20 years of age at the start of chemotherapy, with an Eastern Cooperative Oncology Group performance status of 0–1; and (c) adequate bone marrow reserves and renal/liver functions. Further, the *KRAS* wt or *RAS* wt status was considered in patients treated with cetuximab, while these statuses were neglected when treated with bevacizumab. The investigators evaluated the tumour response using computed tomography based on the guidelines of the response evaluation criteria in solid tumours (RECIST, version 1.1). The ORR was defined as the sum of complete and partial response, the PFS was measured from the start of chemotherapy until the first documented progression or death, while the OS was measured from the date of commencing chemotherapy until death or the date of last evaluation. Primary tumours originating in the splenic flexure, descending colon, sigmoid colon or rectum were classified as left-sided mCRC, whereas, those originating in the cecum, ascending colon, hepatic flexure or transverse colon were classified as right-sided mCRC. ETS was defined as the relative change in the sum of the longest diameters of the RECIST target lesions from baseline till the 8th Week, and a 20% decrease was set as the cut-off value to discriminate early responders (ETS ( +)) from non-responders (ETS (−)) as described previously^3,20,33^.

DpR was defined as the percentage change in the sum of the longest diameters of the RECIST target lesions at the nadir in the absence of new lesions or the progression of non-target lesions compared to the baseline^3,19,20^. DpR was evaluated as a continuous variable (i.e., each patient’s maximum percentage of shrinkage). DpR had a negative value for tumour reduction, positive for tumour growth, and zero for no change. These median values were set as cut-off points. The last follow-up date was August 25, 2020.

## Statistical analysis

The Kaplan–Meier method was used to estimate PFS and OS, and these were compared with the log-rank test. A Cox proportional hazard model was used to calculate the hazard ratios (HR) and 95% confidence intervals (CI) for PFS and OS regarding the interaction between tumour origin (right or left), treatment (chemotherapy plus cetuximab or chemotherapy plus bevacizumab), ETS, and DpR as explanatory variables.

Fisher’s exact test was used to analyse categorical variables of patient characteristics in 2 × 2 contingency tables. All statistical analyses were performed using Ekuseru–Toukei 2015 (Social Survey Research Information Co., Tokyo, Japan). Although these were post hoc analyses, a two-sided *p* value < 0.05 was indicative of notable differences and considered statistically significant.

## Supplementary information

Supplementary Informations.

Supplementary Legends.
