# Supplementary material for: Clinical impact of primary tumour location, early tumour shrinkage, and depth of response in the treatment of metastatic colorectal cancer with first-line chemotherapy plus cetuximab or bevacizumab
Source: Sci Rep. 2020 Nov 13;10:19815. doi: 10.1038/s41598-020-76756-1 (PMC7666202; doi:10.1038/s41598-020-76756-1)
Supplement: Supplementary file 1 — Supplementary Informations. [file 41598_2020_76756_MOESM1_ESM.pptx]

## Slide 1
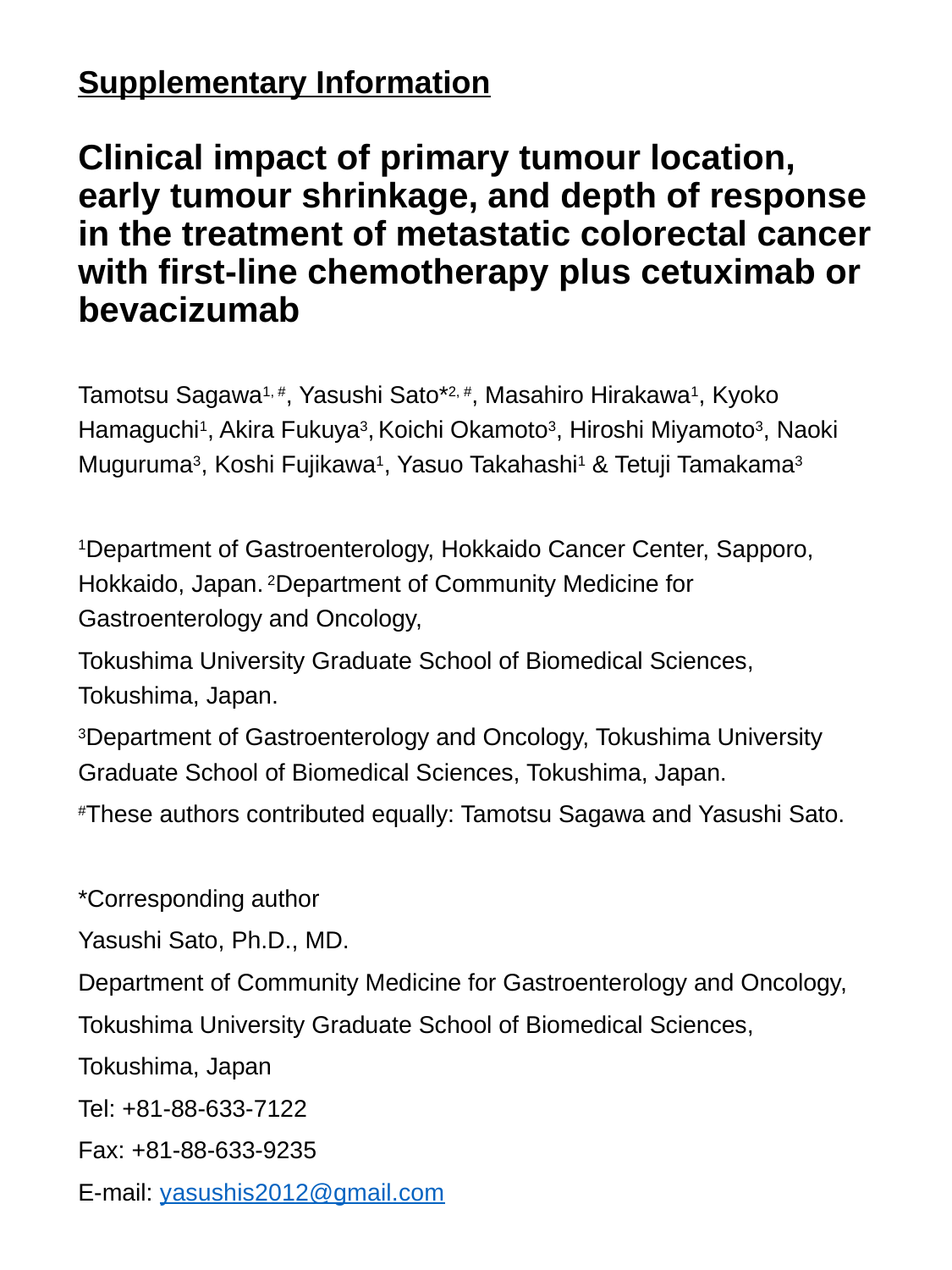

# Supplementary Information Clinical impact of primary tumour location, early tumour shrinkage, and depth of response in the treatment of metastatic colorectal cancer with first-line chemotherapy plus cetuximab or bevacizumab
Tamotsu Sagawa1, #, Yasushi Sato*2, #, Masahiro Hirakawa1, Kyoko Hamaguchi1, Akira Fukuya3, Koichi Okamoto3, Hiroshi Miyamoto3, Naoki Muguruma3, Koshi Fujikawa1, Yasuo Takahashi1 & Tetuji Tamakama3
1Department of Gastroenterology, Hokkaido Cancer Center, Sapporo, Hokkaido, Japan. 2Department of Community Medicine for Gastroenterology and Oncology,
Tokushima University Graduate School of Biomedical Sciences, Tokushima, Japan.
3Department of Gastroenterology and Oncology, Tokushima University Graduate School of Biomedical Sciences, Tokushima, Japan.
#These authors contributed equally: Tamotsu Sagawa and Yasushi Sato.
*Corresponding author
Yasushi Sato, Ph.D., MD.
Department of Community Medicine for Gastroenterology and Oncology,
Tokushima University Graduate School of Biomedical Sciences,
Tokushima, Japan
Tel: +81-88-633-7122
Fax: +81-88-633-9235
E-mail: yasushis2012@gmail.com

## Slide 2
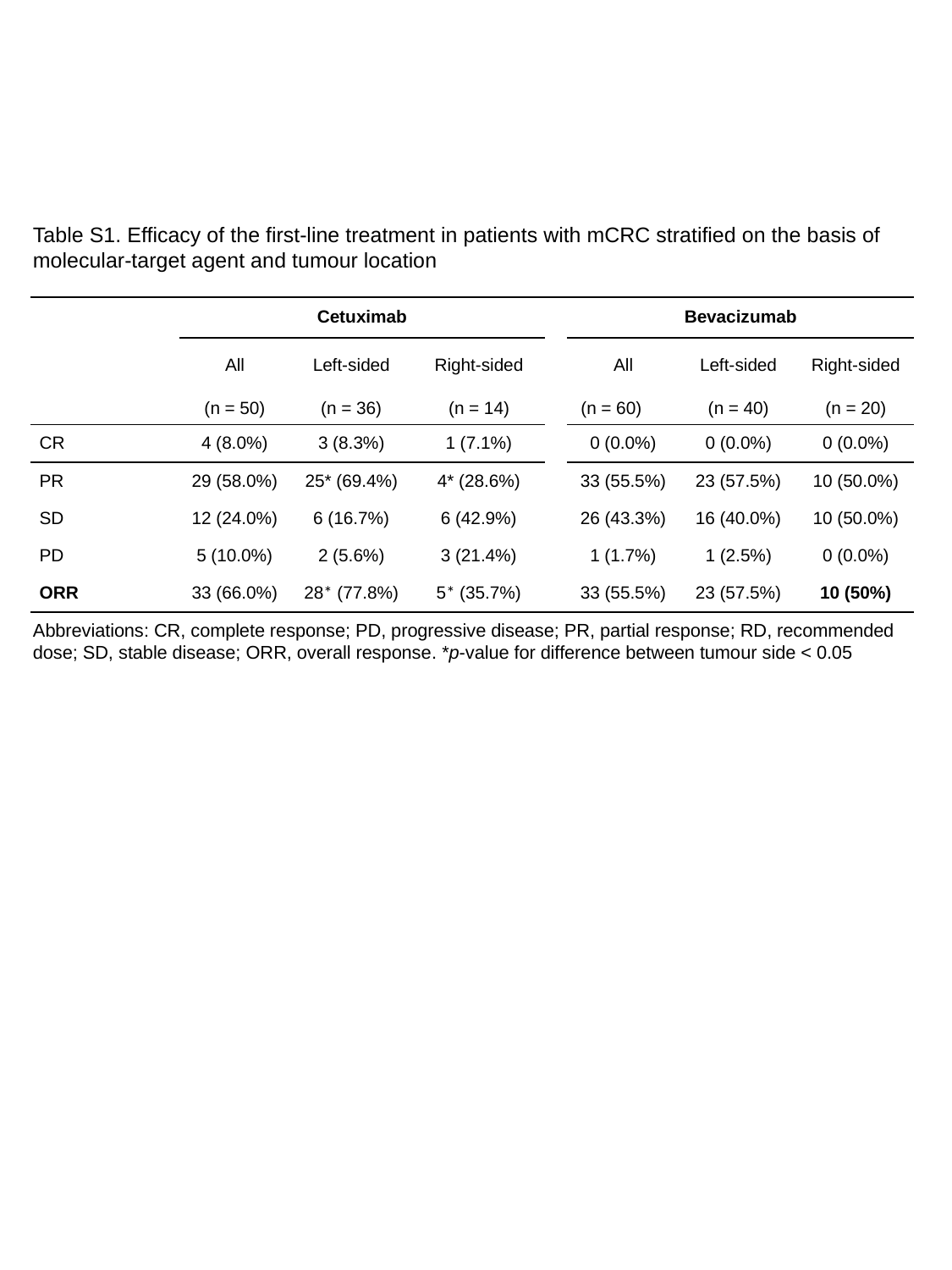

Table S1. Efficacy of the first-line treatment in patients with mCRC stratified on the basis of molecular-target agent and tumour location
| | Cetuximab | | | | Bevacizumab | | |
| --- | --- | --- | --- | --- | --- | --- | --- |
| | All | Left-sided | Right-sided | | All | Left-sided | Right-sided |
| | (n = 50) | (n = 36) | (n = 14) | | (n = 60) | (n = 40) | (n = 20) |
| CR | 4 (8.0%) | 3 (8.3%) | 1 (7.1%) | | 0 (0.0%) | 0 (0.0%) | 0 (0.0%) |
| PR | 29 (58.0%) | 25\* (69.4%) | 4\* (28.6%) | | 33 (55.5%) | 23 (57.5%) | 10 (50.0%) |
| SD | 12 (24.0%) | 6 (16.7%) | 6 (42.9%) | | 26 (43.3%) | 16 (40.0%) | 10 (50.0%) |
| PD | 5 (10.0%) | 2 (5.6%) | 3 (21.4%) | | 1 (1.7%) | 1 (2.5%) | 0 (0.0%) |
| ORR | 33 (66.0%) | 28\* (77.8%) | 5\* (35.7%) | | 33 (55.5%) | 23 (57.5%) | 10 (50%) |
Abbreviations: CR, complete response; PD, progressive disease; PR, partial response; RD, recommended dose; SD, stable disease; ORR, overall response. *p-value for difference between tumour side < 0.05

## Slide 3
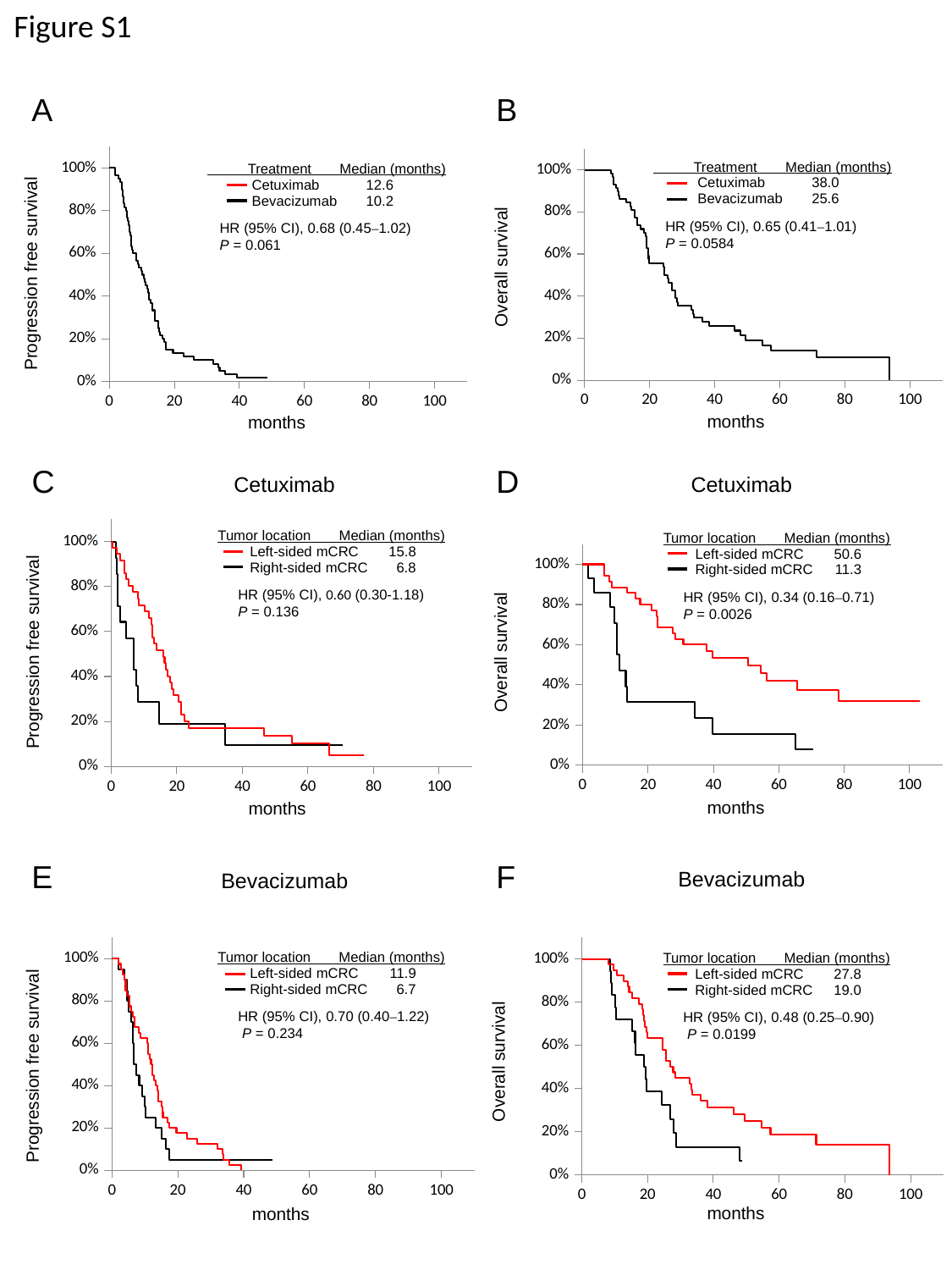

Figure S1
A
B
### Chart
| Category | 系列1 | 系列2 |
|---|---|---|
### Chart
| Category | 系列1 | 系列2 |
|---|---|---| Treatment Median (months)
 Cetuximab	38.0
 Bevacizumab	25.6
 HR (95% CI), 0.65 (0.41‒1.01)
 P = 0.0584
 Treatment Median (months)
 Cetuximab	12.6
 Bevacizumab	10.2
 HR (95% CI), 0.68 (0.45‒1.02)
 P = 0.061
Overall survival
Progression free survival
months
months
C
D
Cetuximab
Cetuximab
### Chart
| Category | 系列1 | 系列2 |
|---|---|---|Tumor location Median (months)
 Left-sided mCRC	15.8
 Right-sided mCRC	6.8
 HR (95% CI), 0.60 (0.30-1.18)
 P = 0.136
Tumor location Median (months)
 Left-sided mCRC	50.6
 Right-sided mCRC	11.3
 HR (95% CI), 0.34 (0.16‒0.71)
 P = 0.0026
### Chart
| Category | 系列1 | 系列2 |
|---|---|---|Progression free survival
Overall survival
months
months
E
F
Bevacizumab
Bevacizumab
### Chart
| Category | 系列1 | 系列2 |
|---|---|---|
### Chart
| Category | 系列1 | 系列2 |
|---|---|---|Tumor location Median (months)
 Left-sided mCRC	11.9
 Right-sided mCRC	6.7
 HR (95% CI), 0.70 (0.40‒1.22)
 P = 0.234
Tumor location Median (months)
 Left-sided mCRC	27.8
 Right-sided mCRC	19.0
 HR (95% CI), 0.48 (0.25‒0.90)
 P = 0.0199
Overall survival
Progression free survival
months
months
