# Supplementary material for: Clinical impact of primary tumour location, early tumour shrinkage, and depth of response in the treatment of metastatic colorectal cancer with first-line chemotherapy plus cetuximab or bevacizumab
Source: Sci Rep. 2020 Nov 13;10:19815. doi: 10.1038/s41598-020-76756-1 (PMC7666202; doi:10.1038/s41598-020-76756-1)
Supplement: Supplementary file 2 — Supplementary Legends. [file 41598_2020_76756_MOESM2_ESM.docx]

**Supporting information**

**Table S1.** Efficacy of the first-line treatment in patients with mCRC stratified based on the molecular-target agent and tumour location.

**Figure S1. Kaplan–Meier analyses of mCRC samples based on the molecular-target agent.** (A) PFS and (B) OS for all the patients; (C) PFS in patients with chemotherapy plus cetuximab based on primary tumour location; (D) OS in patients with chemotherapy plus cetuximab based on primary tumour location; (E) PFS in patients with chemotherapy plus bevacizumab based on primary tumour location; and (F) OS in patients with chemotherapy plus bevacizumab based on tumour location. Abbreviations: CI, confidence interval; HR, hazard ratio.
